# Trends in the uptake of voluntary counselling and testing for HIV in rural Tanzania in the context of the scale up of antiretroviral therapy

**DOI:** 10.1111/j.1365-3156.2011.02877.x

**Published:** 2012-07-30

**Authors:** Raphael Isingo, Alison Wringe, Jim Todd, Mark Urassa, Doris Mbata, Griter Maiseli, Rose Manyalla, John Changalucha, Julius Mngara, Ester Mwinuka, Basia Zaba

**Affiliations:** 1 National Institute of Medical ResearchMwanza, Tanzania; 2 London School of Hygiene and Tropical MedicineLondon, UK

**Keywords:** voluntary counselling and testing, HIV, Tanzania, cohort study

## Abstract

**Objectives:**

To describe trends in voluntary counselling and testing (VCT) use and to assess whether high-risk and infected individuals are receiving counselling and learning their HIV status in rural Tanzania.

**Methods:**

During two rounds of linked serological surveys (2003–2004 and 2006–2007) with anonymous HIV testing among adults, VCT was offered to all participants. The crude and adjusted odds ratios for completing VCT in each survey were calculated to compare uptake by demographic, behavioural and clinical characteristics, stratified by sex. Repeat testing patterns were also investigated.

**Results:**

The proportion of participants completing VCT increased from 10% in 2003–2004 to 17% in 2006–2007, and among HIV-infected persons from 14% to 25%. A higher proportion of men than women completed VCT in both rounds, but the difference declined over time. Socio-demographic and behavioural factors associated with VCT completion were similar across rounds, including higher adjusted odds of VCT with increasing numbers of sexual partners in the past 12 months. The proportion having ever-completed VCT reached 26% among 2006–2007 attendees, with repeat testing rates highest among those aged 35–44 years. Among 3923 participants attending both rounds, VCT completion in 2006–2007 was 17% among 3702 who were HIV negative in both rounds, 19% among 124 who were HIV infected in both rounds and 22% among 96 who seroconverted between rounds.

**Conclusion:**

VCT services are attracting HIV-infected and high-risk individuals. However, 2 years after the introduction of antiretroviral therapy, the overall uptake remains low. Intensive mobilisation efforts are needed to achieve regular and universal VCT use.

## Introduction

In sub-Saharan Africa, where the HIV epidemic has had its most devastating impact, the expansion of voluntary HIV testing and counselling services (VCT) is seen as a key component of public health efforts to lower HIV incidence. VCT aims to provide people with an opportunity to learn and accept their HIV status in a confidential environment, with counselling to encourage sexual behaviour change to prevent further infections (WHO 2005). The scaling up of VCT services could contribute towards reducing HIV transmission if counselling influenced the behaviour of HIV-positive individuals, including those who are sexually active and not yet ill, and HIV-negative individuals with high-risk behaviours.

VCT is also the main entry point for HIV treatment and care services (De Cock *et al.* 2003; Nsigaye *et al.* 2009). In many countries, HIV testing uptake has grown rapidly with the expansion of antiretroviral treatment (ART) – in Tanzania, the proportion ever tested increased from 15% to 32% between 2004 and 2007 (TACAIDS 2005, 2008). However, trends in VCT testing rates among HIV-infected individuals at the population level have rarely been reported, because there are very few settings in which an individual’s HIV status is known prior to VCT. Furthermore, understanding the extent to which VCT already attracts recent seroconverters could provide useful information for the potential development of ‘treatment as prevention’ programs, which would need to identify persons with recent infection to start on ART.

We have previously reported socio-demographic, behavioural and clinical characteristics associated with VCT use among residents of a HIV cohort study in rural Tanzania 2003–2004, shortly before free ART services (Wringe *et al.* 2008). The study showed that although HIV infection and certain high-risk behaviours prompted VCT use, only 10% of individuals completed VCT. VCT was offered free of charge on a fully voluntary basis and was available to study participants in their village of residence, suggesting that VCT uptake was limited by demand factors rather than availability. The purpose of this study is to describe trends in VCT uptake, lifetime VCT coverage and repeat testing patterns from 2004 to 2007 for this rural population-based HIV cohort in northern Tanzania and to investigate whether VCT completion and its determinants have changed since the introduction of free ART in 2005 through the Tanzanian government’s national HIV care and treatment plan.

## Methods

### Study setting

The study was conducted in Kisesa ward, Mwanza region in north-west Tanzania. The ward lies 20 km east of the regional capital, Mwanza City, along the main road to Kenya. Two villages (one of which is a trading centre) lie on this main road, while the other five are rural. The population is served by a health centre on the main road and by three small dispensaries. VCT services have been available at the health centre since 2005, providing referral for those diagnosed with HIV to Mwanza city hospitals, where free care and ART including services for the prevention of mother-to-child transmission of HIV (PMTCT) for pregnant women have been available since 2005 under the national programme. In 2008, ART and PMTCT provision started at Kisesa Health Centre and local patients were transferred there from the city hospitals.

The entire population of the ward is enumerated every 6 months as part of an ongoing Demographic Surveillance System. The population of the ward grew from around 19 000 in 1994, when surveillance started, to about 28 000 in 2007 (Urassa *et al.* 2010). Five serological surveys have been carried out to date in 1994–1995, 1997–1998, 2000–2001, 2003–2004 and 2006–2007. During these serosurveys, adults aged 15 years and above, who constitute about 50% of the total population, provided blood samples for anonymous HIV testing and completed detailed survey questionnaires covering sexual behaviour, use of health services and attitudes towards HIV (Wambura *et al.* 2007). The serological and behavioural surveillance data are anonymously linked to information collected through the demographic surveillance rounds. In each serological survey, all resident adults were invited to come to a temporary clinic located at a central place in each village; since the 2000–2001 round, a separate VCT service has been provided for those opting to know their HIV results.

### Voluntary counselling and testing services during the serosurvey

As VCT uptake at the 2000–2001 round was <1%, this analysis uses data from the 2003–2004 and 2006–2007 rounds. In the 2003–2004 and 2006–2007 surveys, participants were asked if they would like to use the survey VCT service to know their HIV status. Those who agreed were directed to a trained counsellor in a separate hut for pre-and post-test counselling. In 2003–2004, venous blood was collected by the counsellors and transported to the NIMR laboratory for HIV testing, and clients returned for the results 1 week later. In 2006–2007, venous blood was again collected, but rapid HIV screening tests were used - a preliminary test using Capillus, confirmed by the Determine test for those testing positive. Quality control was performed at the NIMR laboratory on a 5% sub-sample using ELISA tests as previously.

Individuals who underwent VCT in the serological survey in 2003–2004 and 2006–2007 were found to be HIV negative and were encouraged to repeat VCT regularly. Those who discovered they were positive in 2003–2004 were informed that treatment would become available in the near future and (with their prior agreement) subsequently traced by the VCT counsellors after the start of the ART programme and referred to the zonal hospital for HIV care and treatment. Those who were positive in 2006–2007 were referred directly to the hospital for HIV care and treatment. Bus fares and a community volunteer were provided to escort persons to the hospital if they wished (Nsigaye *et al.* 2009).

### Reported use of VCT services

The survey questionnaire captures information on participants’ previous use of HIV testing services, including the place and circumstances of HIV testing and whether the results were received and post-test counselling was given. In the 2006–2007 round, participants who reported previous VCT use were also asked about their experiences of using these services.

### Risk behaviour

Survey respondents were asked about their sexual behaviour, including frequency of intercourse, number of sexual partners in the last 12 months, and condom use with spouse, regular and casual partners. Bar girls are defined as ‘high risk’ partners for men, and regular travellers (traders, truck drivers and fishermen) are regarded as ‘high risk’ partners for women – extra questions were asked to probe for these kinds of partners.

### Data analysis

All survey data were double entered, and linking of interview data, HIV research tests and VCT attendance was carried out anonymously using study identification numbers. Frequencies and crude associations were obtained using cross-tabulations and Chi-square tests. To identify factors independently associated with VCT uptake, multivariable logistic regression models were constructed using a forward-fitting approach that considered socio-demographic factors first, followed by behavioural factors to identify potential confounders. HIV status was included in the models as an *a priori* risk factor of interest. Likelihood ratio tests were used to assess the inclusion of variables in the model. Analyses were carried out separately for men and women to assess whether patterns of VCT use differ between the sexes. Data were analysed using Stata 10.

### Ethical approval

Ethical approval for all surveys was given by the Medical Research Coordinating Committee of the Tanzanian Ministry of Health and by the London School of Hygiene and Tropical Medicine.

## Results

### VCT uptake at the survey rounds

**Figure 1:**
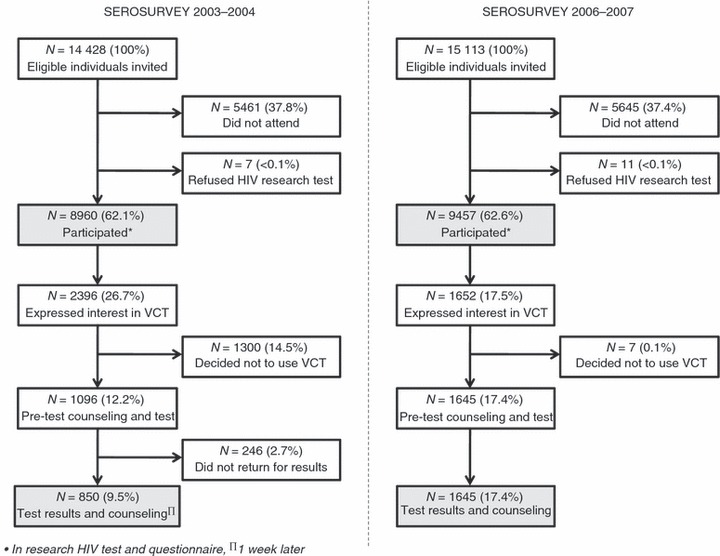
Participation rates, and Voluntary counselling and testing (VCT) interest, pre-test counselling and completion rates in the 2003/2004 and 2006/2007 surveys. **The percentage expressing interest in VCT, undergoing pre-test counselling and HIV test, and completing VCT are calculated among the individuals participating in the survey.

In 2003–2004, 8960 individuals were seen (62.1% of eligible adult residents), of whom 4990 (56%) were women. In 2006–2007, 9457 individuals were seen (62.6% of eligible adult residents), of whom 5572 (59%) were women (Figure 1). Further characteristics of the participants are summarised in Table 1.

**Table 1:** Distribution of voluntary counselling and testing (VCT) uptake by socio-demographic characteristics and reported behavioural factors in 2003–2004 and 2006–2007

		2003–2004				2006–2007			
			
		Males		Females		Males		Females	
					
			VCT Uptake		VCT Uptake		VCT Uptake		VCT Uptake
									
Variable	Category	Total	*n* (%)	Total	*n* (%)	Total	*n* (%)	Total	*n* (%)
All		3970	482 (12)	4990	368 (7)	3885	751 (19)	5572	894 (16)
Age	15–24	1665	159 (10)	1700	142 (8)	1709	234 (14)	1938	299 (15)
	25–34	901	150 (17)	1291	125 (10)	760	223 (29)	1411	304 (22)
	35–44	595	100 (17)	892	75 (8)	521	136 (26)	860	169 (20)
	45+	849	73 (9)	1107	26 (2)	895	158 (18)	1363	122 (9)
Residence	Rural	2298	238 (10)	2616	133 (5)	2341	403 (17)	3056	397 (13)
	Roadside	801	98 (12)	1108	106 (10)	834	194 (23)	1195	251 (21)
	Trading Centre	871	146 (17)	1266	129 (10)	710	154 (22)	1321	246 (19)
	Married Monogamous	1944	250 (13)	2624	185 (7)	1763	395 (22)	2680	461 (17)
Marital status	Single/never married	1597	165 (10)	796	66 (8)	1739	250 (14)	1144	144 (13)
	Married polygamous	139	22 (16)	532	45 (8)	132	42 (32)	588	121 (21)
	Separated/widowed	290	45 (16)	1038	72 (7)	251	64 (25)	1160	168 (14)
Education	None	623	25 (4)	1663	40 (2)	713	103 (14)	2173	248 (11)
	Primary 1–4	791	73 (9)	789	56 (7)	484	74 (15)	466	75 (16)
	Primary 5–7	2214	312 (14)	2342	236 (10)	2146	432 (20)	2557	490 (19)
	Secondary	342	72 (21)	196	36 (18)	542	142 (26)	376	81 (22)
Religion	Christian	2953	417 (14)	4315	332 (8)	2251	404 (18)	2744	444 (16)
	Muslim	105	17 (16)	154	26 (17)	100	25 (25)	165	34 (21)
	Traditional	912	48 (5)	521	10 (2)	1534	322 (21)	2663	416 (16)
Ethnic group	Sukuma	3766	435 (12)	4602	316 (7)	3688	695 (19)	5193	810 (16)
	non-Sukuma	204	47 (23)	388	52 (13)	197	56 (28)	379	84 (22)
Ever had sex	No	385	7 (2)	280	5 (2)	598	36 (6)	635	38 (6)
	Yes	3585	475 (13)	4710	363 (8)	3287	715 (22)	4937	856 (17)
Sex with high-risk partner & condom use in last 12 months	No high-risk partner	3817	448 (12)	4761	325 (7)	3693	699 (19)	5117	785 (15)
	Yes, condom used	52	15 (29)	45	17 (38)	30	7 (23)	53	21 (40)
	Yes, no condom used	101	19 (19)	184	26 (14)	162	45 (28)	402	88 (22)
Number of sexual partners in last 12 months	None	601	24 (4)	884	25 (3)	930	90 (10)	1578	117 (7)
	One	1720	199 (12)	3513	271 (8)	2194	464 (21)	3668	692 (19)
	Two	740	113 (15)	380	30 (8)	484	122 (25)	245	60 (24)
	Three or more	901	146 (16)	213	42 (20)	277	75 (27)	81	25 (31)
Had STI in last 12 months	No	3472	408 (12)	4510	324 (7)	3747	724 (19)	5063	772 (15)
	Yes	498	74 (15)	480	44 (9)	138	27 (20)	509	122 (24)
HIV Status	HIV−	3739	441 (12)	4685	334 (7)	3690	691 (19)	5180	806 (16)
	HIV+	231	41 (18)	305	34 (11)	195	60 (31)	392	88 (22)

In 2003–2004, 27% (2396/8960) of participants expressed a desire for VCT with a higher proportion among men than women (30%*vs.* 24%); 12% (1096/8960) underwent pre-test counselling and HIV testing, and 10% (850/8960) returned a week later for test results and post-test counselling. In 2006–2007, 18% (1652/9457) of participants indicated an interest in receiving VCT (19% of men and 16% of women) and 17% (1645/9457) subsequently underwent pre-test counselling, HIV testing and received their results with post-test counselling, representing a 70% increase in VCT completion from the previous survey round.

Among men and women, the biggest increases in VCT completion between the two surveys were for those aged 45 and over, those in polygamous marriages, those with no schooling and those following traditional religions (Table 1). Among women only, VCT uptake increased by over 150% among residents of remote rural villages by the 2006–2007 round.

Factors associated with VCT uptake in 2003–2004 have been described in detail elsewhere (Wringe *et al.* 2008) and are presented in Table 2. In brief, for both sexes, factors independently and positively associated with completing VCT included higher education, being from a non-Sukuma tribe and reporting three or more sexual partners in the last 12 months. Among both sexes, the adjusted odds of VCT use were lower among those following a traditional religion, compared to Christians or Muslims, and being 45 years or above. For women only, living in a roadside village and using a condom with a high-risk partner in the last 12 months were associated with higher VCT use. For men only, the adjusted odds of VCT use were higher among HIV infected than those who were HIV negative.

**Table 2:** Crude and adjusted* odds ratios for factors associated with voluntary counselling and testing uptake among survey participants in 2003–2004, by sex

		Male (*N* = 3970)				Female (*N* = 4990)			
		Crude OR 95% CI		Adjusted OR (95%CI)		Crude OR 95% CI		Adjusted OR (95%CI)	
Variables	Category								
Age (years)	15–24	1		1		1		1	
	25–34	1.84	1.45, 2.34	1.25	0.91, 1.71	1.18	0.91, 1.51	1.08	0.81, 1.44
	35–44	1.86	1.42, 2.44	1.32	0.90, 1.92	1.01	0.75, 1.35	1.19	0.85, 1.67
	45+	0.87	0.65, 1.16	0.92	0.61, 1.39	0.26	0.17, 0.40	0.50	0.29, 0.85
Residence	Rural	1				1		1	
	Road side	1.21	0.94, 1.55	0.99	0.76, 1.28	1.97	1.51, 2.58	1.67	1.27, 2.21
	Trading Centre	1.74	1.39, 2.18	1.17	0.91, 1.49	2.12	1.65, 2.73	1.31	0.98, 1.72
Marital status	Married Monogamous	1		1		1		1	
	Single-never married	1.28	1.04, 158	0.95	0.69, 1.31	1.19	0.89, 1.60	1.19	0.83, 1.71
	Married polygamous	1.63	1.01, 2.65	1.47	0.88, 248	1.22	0.87, 1.71	1.41	0.99, 2.02
	Separated or widowed	1.59	1.12, 2.28	1.34	0.92, 1.95	0.98	0.74, 1.30	1.52	1.08, 213
Education	None	1		1		1		1	
	Primary: 1–4 years	2.43	1.52, 3.88	2.04	1.25, 3.26	3.10	2.05, 4.69	2.38	1.54, 3.67
	Primary: 5–7 years	3.92	2.58, 5.96	2.76	1.75, 4.27	4.55	3.23, 6.40	3.06	2.09, 4.58
	Secondary+	6.38	3.96, 10.28	4.03	2.40, 6.77	9.13	5.66, 14.73	6.02	3.50, 10.37
Religion	Christian	1		1		1		1	
	Islam	1.17	0.69, 1.99	0.99	0.57, 1.72	2.44	1.58, 3.77	2.05	1.28, 3.29
	Traditional	0.34	0.25, 0.46	0.47	0.34, 0.66	0.23	0.12, 0.44	0.47	0.24, 0.90
Ethnic group	Sukuma	1		1		1		1	
	Non-Sukuma	2.29	1.63, 3.22	1.65	1.14, 2.39	2.10	1.53, 2.87	1.35	0.96, 1.91
								
Ever had sex	No	1		1		1		1	
	Yes	8.25	3.88, 17.53	3.72	1.49, 9.31	4.59	1.88, 11.19	4.00	1.37, 11.68
Sex with high-risk partner & condom use in last 12 months	No high-risk partner	1		1		1		1	
	Yes, condom used	3.05	1.66, 5.60	1.65	0.86, 3.13	8.30	4.50, 15.30	3.46	1.70, 7.10
	Yes, no condom used	1.74	1.05, 2.90	1.42	0.82, 2.46	2.25	1.46, 3.45	1.16	0.71, 1.89
Number of sexual partners in last 12 months	None	1		1		1		1	
	One	3.14	2.04, 4.85	1.66	0.96, 2.87	2.87	1.89, 4.36	1.44	0.82, 2.51
	Two	4.33	2.75, 6.83	2.02	1.14, 3.55	2.95	1.71, 5.08	1.19	0.61, 2.31
	Three+	4.60	2.95, 7.18	2.14	1.21, 3.72	8.44	5.00, 14.22	2.76	1.40, 5.45
Had STI in last 12 months	No	1		1		1		1	
	Yes	1.31	1.00, 1.71	1.11	0.83, 1.48	1.30	0.94, 1.81	1.07	0.75, 151
HIV Status	HIV−	1		1		1		1	
	HIV+	1.61	1.13, 2.30	1.42	0.99, 2.05	1.63	1.12, 2.37	1.21	0.81, 1.79

* Odds ratios adjusted for all other variables.

In the 2006–2007 survey (Table 3), the adjusted analysis showed that the uptake of VCT remained strongly associated with higher levels of education in both men (aOR = 2.59; 95% CI: 1.86–3.61) and women (aOR = 2.27; 95% CI:1.62–3.18) and living in the roadside areas for men (aOR = 1.39; 95% CI: 1.13–1.70) and women (aOR = 1.81; 95% CI: 1.51–2.17). Adjusted odds ratios of VCT uptake were also higher among the polygamously married males (aOR = 1.73; 95% CI: 1.12–2.67) and females (aOR = 1.41; 95% CI: 1.11–1.77) and for separated or widowed males (aOR = 1.44; 95% CI; 1.03–2.00). VCT uptake remained lower among women aged 45 years or more (aOR = 0.62; 95%CI: 0.46–0.83) and among men and women who followed traditional religions (aOR = 0.76; 95%CI: 0.60–0.97 and aOR = 0.63; 95%CI: 0.44–0.91, respectively).

**Table 3:** Crude and adjusted* odds ratios for factors associated with voluntary counselling and testing uptake among 2006–2007 survey participants, by sex

		Male (*N* = 3885)				Female (*N* = 5572)			
			
		Crude OR 95% CI		Adjusted OR (95%CI)		Crude OR 95% CI		Adjusted OR (95%CI)	
Variables	Category								
Age (years)	15–24	1		1		1		1	
	25–34	2.62	2.13, 3.22	1.78	1.30, 2.43	1.51	1.26, 1.80	1.11	0.90, 1.36
	35–44	2.23	1.75, 2.83	1.43	0.99, 2.06	1.34	1.09, 1.65	1.03	0.81, 1.31
	45+	1.35	1.08, 1.68	1.02	0.70, 1.46	0.54	0.43, 0.67	0.62	0.46, 0.83
Residence	Rural	1		1		1		1	
	Roadside	1.47	1.21, 1.78	1.39	1.13, 1.70	1.78	1.50, 2.12	1.81	1.51, 2.17
	Trading centre	1.34	1.08, 1.65	1.12	0.89, 1.42	1.53	1.29, 1.82	1.31	1.08, 1.58
Marital status	Married Monogamous	1		1		1		1	
	Single-never married	1.72	1.44, 2.05	0.81	0.59, 1.11	0.69	0.57, 0.85	0.99	0.75, 1.31
	Married polygamous	2.78	1.88, 4.11	1.73	1.12, 2.67	1.25	1.00, 1.56	1.41	1.11, 1.77
	Separated or widowed	2.04	1.49, 2.79	1.44	1.03, 2.00	0.82	0.67, 0.99	1.57	1.24, 2.00
Education	None	1		1		1		1	
	Primary: 1–4 years	1.07	0.77, 1.48	1.03	0.73, 1.43	1.49	1.13, 1.97	1.22	0.91, 1.63
	Primary: 5–7 years	1.50	1.18, 1.89	1.45	1.12, 1.87	1.84	1.56, 2.17	1.54	1.28, 1.86
	Secondary or more	2.11	1.58, 2.79	2.59	1.86, 3.61	2.13	1.61, 2.82	2.27	1.62, 3.18
Religion	Christian	1		1		1		1	
	Islam	1.52	0.96, 2.43	1.1	0.68, 1.80	1.34	0.91, 1.99	1.09	0.72, 1.65
	Traditional	1.22	1.03, 1.43	0.76	0.60, 0.97	0.96	0.83, 1.11	0.63	0.44, 0.91
Ethnic group	Sukuma	1		1		1		1	
	Non-Sukuma	1.71	1.24, 2.36	1.38	0.98, 1.96	1.54	1.20, 1.98	1.21	0.92, 1.60
Ever had sex	No	1		1		1		1	
	Yes	4.34	3.07, 6.14	2.41	1.51, 3.86	3.30	2.35, 4.61	1.81	1.12, 2.93
Sex with high-risk partner and condom use in last 12 months	No high-risk partner	1		1		1		1	
	Yes, condom used	1.30	0.60, 3.05	1.47	1.01, 2.14	3.62	2.08, 6.31	1.95	1.09, 3.48
	Yes, no condom used	1.65	1.16, 2.34	1.26	0.90, 1.76	1.56	1.21, 1.98	0.96	0.74, 1.25
Number of sexual partners in last 12 months	None	1		1		1		1	
	One	2.50	1.97, 3.18	1.26	0.90, 1.76	2.90	2.36, 3.57	2.19	1.61, 2.97
	Two	3.15	2.33, 4.24	1.40	0.94, 2.08	4.05	2.86, 5.73	2.74	1.81, 4.17
	Three+	3.47	2.46, 4.88	1.72	1.12, 2.64	5.57	3.36, 9.26	3.98	2.26, 7.01
Had STI in last 12 months	No	1		1		1		1	
	Yes	0.98	0.64, 1.51	0.84	0.54, 1.31	0.57	0.46, 0.71	1.47	1.17, 1.84
HIV status	HIV−	1		1		1		1	
	HIV+	1.93	1.41, 2.64	1.43	1.03, 1.99	1.57	1.22, 2.01	1.2	0.93, 1.56

* Odds ratios adjusted for all other variables.

Men and women who reported sex with a high-risk partner in the past year and using a condom were more likely to complete VCT (aOR = 1.47; 95%CI 1.01–2.14; aOR = 1.95; 95 CI: 1.09–3.48, respectively). Likewise, having three or more sexual partners in the last 12 months was independently associated with VCT uptake for men (aOR = 1.72; 95% CI: 1.12–2.64) and women (aOR = 3.98; 95% CI: 2.26–7.01). HIV-infected individuals had higher odds of VCT uptake compared to uninfected men (aOR = 1.43; 95% CI: 1.03–1.99) and women (aOR = 1.2; 95% CI: 0.93–1.56).

### Lifetime coverage with VCT services by 2006–2007

Among the 9457 participants at the 2006–2007 round, 15% (1234/8221) of previously untested persons used VCT services for the first time during the survey, and 411/1236 (33%) of those with documented or reported previous VCT use opted to use these services again, resulting in a substantial increase in VCT coverage as a result of the 2006–2007 survey. Of the 1179 individuals at the 2006–2007 round who reported previous VCT, 548 (46%) reported VCT at the 2003–2004 round (our records show an additional 23 completed VCT at the 2003–2004 round, but did not report this at the next round); 171 (15%) reported using the permanent VCT service at Kisesa health centre; 141 (12%) reported testing with a local NGO provider; 62 (5%) women were tested at ANC as part of PMTCT services, and 252 (21%) had VCT ‘elsewhere’.

**Figure 2:**
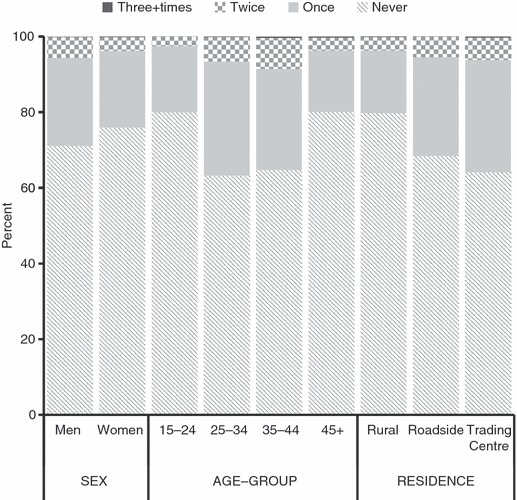
Frequency of reported and documented voluntary counselling and testing by 2006–2007, by sex, age group and residence.

Figure 2 presents lifetime coverage with VCT services by sex, age group and area of residence at the 2006–2007 survey, based on previous VCT use reported through the survey questionnaires, and documented during the serosurveys. The proportion having never used VCT following the 2006–2007 round was highest among women (76%), those aged 45 years and over (80%) and those residing in remote rural villages (80%). Among the 2470 persons who had ever learned their HIV status by the end of the 2006–2007 round, 524 (21%) had used VCT services on more than one occasion, with higher proportions among men (24%) and 35–45 year olds (30%).

### VCT use in the 2006–2007 round by HIV status history

Among the 9457 individuals attending the 2006–2007 survey, 3923 individuals had also attended the 2003–2004 survey. Of these, 3702 (94%) were HIV negative on both occasions, 124 (3%) were HIV positive on both occasions, and 96 (2%) seroconverted during the 3-year period. VCT uptake in 2006–2007 was 17% (623/3702) among the repeat negatives, 19% (24/124) among the repeat positives and 22% (21/96) among those who seroconverted between the two rounds. The proportion of repeat negatives that completed VCT at both rounds was 20% (742/3702), while the proportion of seroconverters that completed VCT at both rounds was 27% (26/96) and the proportion of repeat positives that completed VCT at both rounds was 24% (30/124).

Among the 5534 who *only* attended the 2006–2007 round, 28% of the 367 who were HIV infected used VCT and 17% of the 5167 HIV negative used VCT; 453/587 (77%) HIV-infected persons who attended the 2006–2007 round had not previously completed VCT. Of these, the proportion diagnosed during this survey was 25% (115/453), leaving 58% (338/587) of infected people still unaware of their status.

### Disclosure to partner and attitudes towards VCT services

During each survey, participants reporting previous VCT use were asked about receipt of results, post-test counselling and disclosure of their HIV status to their partner or others (Table 4). Among 81 men and 83 women who reported previous use of the survey HIV testing services in 2003–2004, 74% of men and 61% of women reported receiving post-test counselling. In comparison, 48% of 52 men and 42% of 26 women who reported previous VCT elsewhere at the 2003–2004 round stated that they had received post-test counselling. By 2006–2007, over 80% of men and women reported having received post-test counselling, regardless of place of VCT.

**Table 4:** Voluntary counselling and testing (VCT) completeness and disclosure in 2003–2004 and 2006–2007, and VCT experience in 2006–2007 by sex and place of VCT

	Males		Females	
		
	had VCT in serosurvey	had VCT elsewhere	had VCT in serosurvey	had VCT elsewhere
				
	*n* (%)	*n* (%)	*n* (%)	*n* (%)
*VCT completeness and disclosure in 2003–2004 Total*	81 (100)	52 (100)	83 (100)	26 (100)
Received pre-test counselling in last VCT visit	64 (79)	47 (90)	59 (71)	21 (81)
Received results after last VCT visit	75 (93)	33 (63)	77 (93)	14 (54)
Received post-test counselling in last VCT visit	60 (74)	25 (48)	51 (61)	11 (42)
Revealed results to anyone	66 (81)	27 (52)	74 (89)	11 (42)
*VCT completeness and disclosure in 2006–2007 Total*	259 (100)	290 (100)	366 (100)	258 (100)
Received pre-test counselling in last VCT visit	244 (94)	281 (97)	306 (84)	241 (93)
Received results after last VCT visit	246 (95)	256 (88)	345 (94)	229 (89)
Received post-test counselling in last VCT visit	239 (92)	252 (87)	292 (80)	214 (83)
Revealed results to anyone	144 (56)	139 (48)	249 (68)	140 (54)
*VCT experience in 2006–2007 Total*	259 (100)	290 (100)	366 (100)	358 (100)
Would recommend a friend to have a VCT	241 (93)	271 (93)	346 (95)	237 (92)
VCT counsellor kind and understanding	245 (95)	277 (96)	320 (87)	245 (95)
VCT interview embarrassing	11 (4)	19 (7)	22 (6)	19 (7)
VCT counsellor can be trusted and confidential	229 (88)	249 (86)	256 (70)	182 (70)
Anyone visiting VCT centre assumed to be infected	52 (20)	45 (16)	68 (19)	59 (23)

In 2003–2004, disclosure rates were higher among those reporting prior use of the survey VCT service compared to those using other HIV testing centres (81% of men and 89% of women *vs.* 52% of men and 42% of women). By 2006–2007, overall disclosure rates had slightly declined from the earlier round, with a smaller difference observed by place of VCT.

One thousand one hundred and seventy-nine participants who reported previous VCT use in 2006–2007 were additionally asked their views on service quality: over 90% of men and women reported they would recommend VCT to a friend, regardless of where they had used VCT, and <10% of men and women reported that the VCT session was embarrassing, regardless of where they had used VCT. Trust in the confidentiality of the counsellors was higher among men than women, regardless of the service used: 88% of male users of survey VCT services and 86% of other VCT users, compared with 70% of women who had used either the survey VCT service or other VCT services.

## Discussion

Overall, our study has shown that VCT completion in Kisesa ward was worryingly low at 10% in 2003–2004 and 17% in 2006–2007. The increase in 2006–2007 may be due to factors such as the availability of rapid HIV tests with results given within 15 min of testing, knowledge of the availability of free ART and a national VCT campaign in early 2005. Other studies in African settings have also shown that free access to ART motivates individuals to undergo VCT, but is not sufficient to ensure high rates of testing uptake (Day *et al.* 2003).

Compared to the Tanzanian national picture from the demographic and health surveys (DHS) where 15% men and women had ever tested in 2004 and 37% women and 26% men had ever tested in 2007 (TACAIDS 2005, 2008), the proportion of ever tested in Kisesa is low: 7% of women and 12% men in 2004 rising to 24% of women and 29% of men in 2007. However, the DHS results for rural Mwanza region are much closer to the Kisesa results, which will partly reflect the later introduction of provider-initiating testing and counselling and antenatal testing in many rural areas such as Kisesa (where PMTCT only started at the end of 2008).

The proportion of participants who expressed interest in VCT in 2003–2004 was higher than in 2006–2007 (27%*vs.* 18%), but in terms of those completing the procedure, higher proportions were recorded in 2006–2007 compared to 2003–2004. Introduction of HIV rapid tests could partly explain this. Other possible explanations for the low completion rates in 2003–2004 by those who expressed an interest in VCT include HIV-related stigma, confidentiality concerns and indecision, which is similar to findings from other African studies (Matovu & Makumbi 2007; Bwambale *et al.* 2008). Encouragingly in Kisesa, by 2006–2007, 78% of those who reported prior VCT use stated that the counsellors could be trusted.

Despite variations in VCT completion rates in the two rounds, the socio-demographic characteristics of VCT users were similar. In particular, the strongest predictor of VCT uptake for both sexes in each survey round was higher levels of education, as found in certain other studies (Fabiani *et al.* 2007; Bwambale *et al.* 2008). In both rounds, older participants, those with least schooling, those following traditional religions and women living in the most remote rural villages remained relatively underserved by the VCT services, highlighting the need for extra efforts to reach these groups. Nevertheless, it was encouraging that these inequalities in testing coverage declined over the two rounds. Odds ratios reported in this study do not directly translate into risk ratios for completion of VCT, but can be used to identify inequalities in completion of VCT.

Both surveys provided good opportunities for people to access VCT as the clinics were brought to the villages, enhancing access and most of those who took up the service had never previously undergone VCT. Although the provision of VCT at 2006–2007 almost doubled the proportion of people who had ever undergone VCT in this population, a high proportion of women, older individuals and residents in remote villages have yet to use VCT. Improving access for these groups may require alternative methods for providing VCT-like door-to-door HIV testing services or regular mobile HIV testing clinics (Matovu & Makumbi 2007; Mutale *et al.* 2010).

In terms of high-risk behaviours, VCT use generally increased with higher numbers of sexual partners in the past year for men and women in both rounds. VCT uptake was higher among those reporting sex with a high-risk partner and condom use in the past 12 months. By adjusting for any sexual experience in the multivariable analysis, VCT completion in the group reporting high-risk sexual partners, condom use and higher number of partners, is compared to those who are sexually active but who do not report these behaviours. Consistently, higher rates of VCT use among individuals with these risk behaviours indicate risk awareness, although further research is required to assess whether the VCT counselling sessions result in subsequent reductions in reported risk behaviours among these individuals in future survey rounds.

In both surveys, infected persons of both sexes were more likely to test than the uninfected, with this being more apparent among men than women in both rounds. Nevertheless, with only 25% of HIV-positive individuals taking up VCT services during the later round after ART introduction, more progress is needed to achieve the necessary coverage to enable universal access to HIV treatment. During the 2006–2007 round, 25% (115/453) of HIV-infected persons who were hitherto unaware of their status were diagnosed, but this still left 58% (338/587) of infected people unaware of their status.

Regularly repeated VCT is crucial for any development of ‘treatment as prevention’ interventions that would rely on high rates of regular HIV testing to identify recent seroconverters. In our study, among the 3923 individuals who attended both surveys, the proportion of recent seroconverters who accessed VCT in both rounds (27%) was slightly higher than the proportion of repeat negatives that completed VCT at both rounds (20%).

Although this study has the advantage of being undertaken in a community-based HIV cohort study, it relies heavily on self-reporting by respondents which may lead to reporting bias because of reluctance to disclose sensitive behaviours like non-marital sexual activity. Individuals who accessed VCT were invited to participate in the serosurvey and only informed specifically about the availability of VCT during registration. This may have implications for VCT uptake if some were not prepared to make the decision and if married women felt they needed their husband’s approval (Kranzer *et al.* 2008). Although this may have led to a reduction in VCT access, it might ensure wider participation in the serosurvey, as agreement to VCT would not be seen as implicit in survey participation.

In conclusion, this analysis shows that VCT uptake remains low, even after 2 years of free ART. Although VCT services attract HIV-infected individuals and those with high-risk behaviours, several inequalities in testing coverage persist, especially among older women, those with least education and those following traditional religions. The provision of VCT in temporary huts in villages using rapid tests could have a dramatic impact on the overall uptake of HIV testing and should be implemented in other areas in the country to improve VCT uptake.
